# Inhibiting Ceramide Synthesis Attenuates Hepatic Steatosis and Fibrosis in Rats With Non-alcoholic Fatty Liver Disease

**DOI:** 10.3389/fendo.2019.00665

**Published:** 2019-09-26

**Authors:** Meng Jiang, Chun Li, Qiaoshu Liu, Aimin Wang, Minxiang Lei

**Affiliations:** Department of Endocrinology, Xiangya Hospital of Central South University, Changsha, China

**Keywords:** ceramide, NAFLD, NASH, apoptosis, myriocin

## Abstract

Non-alcoholic fatty liver disease (NAFLD) is one of the most common metabolic disorder diseases, which include a histological spectrum of conditions ranging from simple steatosis to non-alcoholic steatohepatitis (NASH). Dysregulated metabolism of sphingomyelin in the liver plays a critical role in the pathogenesis of NAFLD. Ceramides are central molecules of sphingolipid biosynthesis and catabolism and play an important role in insulin resistance, apoptosis, and inflammation. In addition, apoptosis is a main contributor to the development of NAFLD. This study detected whether the inhibition of ceramide synthesis ameliorated hepatic steatosis and fibrosis in rats with NAFLD. Sprague-Dawley rats were used to establish the NAFLD model. Here, we showed that hepatic ceramide, steatosis, and fibrosis increased in liver tissue from rats with NAFLD. Chronic treatment with myriocin inhibited ceramide and lipid accumulation and improved fibrosis in liver tissue samples of high fat diet (HFD)-fed rats. In addition, hepatic inflammation and apoptosis were markedly ameliorated in HFD-fed rats treated with myriocin. Furthermore, myriocin treatment regulated the expression of pro-apoptosis and anti-apoptosis proteins by inactivating the c-Jun N-terminal kinase (JNK) signaling pathway in the liver of HFD-fed rats. Collectively, ceramide plays an important role in the pathogenesis of NASH and may represent a potential therapeutic strategy to prevent NAFLD.

## Introduction

Non-alcoholic fatty liver disease (NAFLD) is one of the most common metabolic disorder diseases, which include a histological spectrum of conditions ranging from simple steatosis to NASH (1). Hepatic steatosis is thought to be a benign condition, while NASH is a more serious hepatic disorder with varying degrees of inflammation, hepatocyte damage, and progressive fibrosis.

NAFLD is an obesity-related disease that is often accompanied by insulin resistance, hypertension, and dyslipidemia. NAFLD is also defined by the cytotoxic accumulation of lipids, such as ceramide (2, 3). Ceramides are sphingolipid molecules that play an important role in insulin resistance, apoptosis, and inflammation (3–10). Previous studies suggest that the inhibition of *de novo* ceramide synthesis reduced hepatic lipid accumulation in rats (11, 12). Therefore, ceramide may play a role in the development of NAFLD and its progression to NASH. However, it is unclear how ceramide affects the pathogenesis of NASH.

Hepatic apoptosis is considered a prominent feature of NAFLD (13, 14). Furthermore, ceramide can mediate cell death in different models, such as endothelial, fibroblast, lymphoblast, and HeLa cell lines (2, 15, 16). In addition, ceramide regulates apoptosis via its interaction with Bcl-2 family proteins (17). Thus, we reasoned that inhibiting ceramide accumulation would improve NAFLD via the regulation of hepatic apoptosis.

As a molecule at the nexus of sphingolipid metabolism, cermide can be generated through three main pathways, including *de novo* synthesis, sphingomyelin degradation, and generation from sphingosine (18). In addition, ceramide is primarily generated via a *de novo* biosynthetic pathway (19). Serine palmitoyltransferase (SPT) is the key enzyme in the ceramide synthetic pathway. Myriocin is an inhibitor of SPT and can reduce ceramide accumulation *in vivo* and *in vitro*. The present study utilized myriocin to explore the effects of a ceramide synthesis inhibitor on rats with NAFLD.

## Materials and Methods

### Animals

Male Sprague–Dawley rats (4–6 weeks of age) were obtained from the Slack Shanghai Laboratory Animal Co., Ltd. The rats were randomly divided into three groups:

- The Control group (Con) was fed a normal rodent diet.
- The NAFLD control group (NC) was fed a high-fat diet (ca. 60% energy from fat) and was also injected with vehicle.
- The NAFLD + myriocin group (NM) was fed a high-fat diet for 12 weeks and, starting on the fourth week of the diet, was also treated with myriocin for 12 weeks. The rats were intraperitoneally injected with myriocin (0.3 mg/kg, Sigma) every other day.

The experimental protocol was approved by the ethical committee of Central South University.

### Ceramide Analysis

The ceramide content (liver and serum) was determined by liquid chromatography-mass spectrometry as we described before (20). In brief, the liver tissues were weighed and homogenized in 0.5 mL of double distilled water. Then, the homogenate was transferred to a new centrifuge tube for lipid isolation. Next, we collected the lipids (11) and dried them via evaporation. Then, we dissolved the dry lipids in the 50 μL mobile phase. After centrifugation, the 5 μL supernatants were transferred into the liquid chromatography-mass spectrometry system. The flow rate was 0.22 mL/min. In this system, we used a chromatographic column (Hypersil-HyPURITY C18, 150 × 2.1 nm, 5 μm, Thermo, USA). The serum ceramide concentration was detected and expressed as ng/ml serum.

### Metabolic Index Determination

Serum glucose, total cholesterol (TC), triglyceride (TG), free fatty acid (FFA), AST, and ALT levels were determined using the Vitalab Selectra E Sequential multiple analyzer. The commercial radioimmunoassay kits were used to measure the serum insulin concentration.

### Histology and Quantification of Steatosis

The cryostat sections (8 μm) of rat livers were fixed with 4% paraformaldehyde. The sections were stained with hematoxylin and eosin (H&E). Meanwhile, we used frozen sections for Oil Red O staining to observe the hepatic lipid content. In addition, we also used frozen sections for Masson's trichrome staining to observe fibrosis. The TG content in the liver tissue was measured using commercial kits (Sigma-Aldrich, St. Louis, MO). First, the liver tissues were weighed and homogenized in deionized water. Then, the total lipids were extracted, dried, and resuspended in dimethyl sulfoxide. Finally, we detected the lipid concentration according to the manufacturer's instructions.

### Immunofluorescence (IF)

The cryostat sections were fixed with 4% paraformaldehyde and made permeable with 0.3% Triton X-100. Then, the sections were blocked with 5% donkey serum in phosphate buffered saline (PBS) and incubated with primary antibodies (α-SMA, cleaved caspase3, cleaved HARP) overnight at 4°C. The primary antibody against α-SMA was obtained from Sigma-Aldrich Chemicals company, cleaved caspase3 and cleaved HARP antibodies were purchased from Cell Signaling Technology. After incubation, any non-specific binding was washed away with a solution of PBS. Then, the samples were incubated with secondary antibody for 2 h at room temperature. Samples were mounted using DAPI (ThermoFisher Scientific, Waltham, MA, USA) as a nuclear marker. Images were generated using a Zeiss microscope (Zeiss, Jena, Germany). Morphometric analyses were performed using ImageJ software.

### RT-PCR and Western Blot Analysis

For RT-PCR analysis, we isolated the total mRNA from the liver tissue using TRIzol Reagent (Invitrogen, USA) and reverse-transcribed the mRNA into cDNA using the PrimeScript 1st strand cDNA Synthesis Kit (Takara, JAPAN) according the manufacturer's instructions. SYBR Green (Takara, JAPAN) was used to quantify the PCR amplification products. The mRNA expression levels were normalized to GAPDH expression. We used the comparative Ct (threshold cycle) method to calculate the relative gene-expression levels. The primers used in the study were as follows:

Rat TNF-α: sense, 5′- ACCACGCTCTTCTGTCTACTG -3′

antisense, 5′- CTTGGTGGTTTGCTACGAC -3′

Rat IL-1β: sense, 5′- GCAATGGTCGGGACATAGTT -3′

antisense, 5′- AGACCTGACTTGGCAGAGGA -3′

Rat IL-6: sense, 5′- TCTCTCCGCAAGAGACTTCCA -3′

antisense, 5′- ATACTGGTCTGTTGTGGGTGG -3′

Rat GAPDH: sense, 5′- AGACAGCCGCATCTTCTTGT -3′

antisense, 5′- CTTGCCGTGGGTAGAGTCAT -3′

For Western blot analysis, the liver tissues were homogenized in RIPA lysis buffer (Beyotime Biotechnology) with a protease inhibitor cocktail (Sigma-Aldrich). Then, the samples were centrifuged (14,000 rpm, 4°C), and the supernatant was collected. The protein concentration was determined by the bicinchoninic acid assay method. Equal amounts of protein samples (50 μg) were loaded into lanes of 12% acrylamide SDS gel. We electrophoresed the gels and transferred the protein to a polyvinylidene fluoride membrane. Then, the membrane was incubated with primary antibodies (cleaved caspase3/ Caspase3, cleaved HARP/HARP, p-JNK/JNK, Cytochrome c, Bcl-2, and Bax). The primary antibodies against Bcl-2 and Bax were purchased from Abcam Biotechnology company, GAPDH antibody was purchased from Santa Cruz Biotechnologies, cleaved caspase3/caspase3, cleaved HARP/HARP, p-JNK/JNK, and Cytochrome c antibodies were purchased from Cell Signaling Technology. Next, we incubated the immunoblots with secondary antibodies. The protein signals were visualized using ECL Western blotting detection reagents (ThermoFisher Scientific, Waltham, MA, USA). The density analysis was quantified with ImageJ Software, and the respective protein expression levels were normalized to the housekeeping proteins GAPDH.

### Statistical Analysis

Data are expressed as the mean ± SEM. Statistical analyses of the results were performed using one-way analysis of variance (ANOVA), and the homogeneity of variances was tested. If heterogeneity of variance was present, group differences were determined by the Game–Howell test; if homoscedasticity was present, group differences were determined by the Student-Newman-Keuls test. The threshold for significance was set at *P* ≤ 0.05. Analyses were performed using SPSS 13.0 software.

## Results

### Effect of Myriocin on Ceramide Content and Metabolic Indices

**Figure 1:**
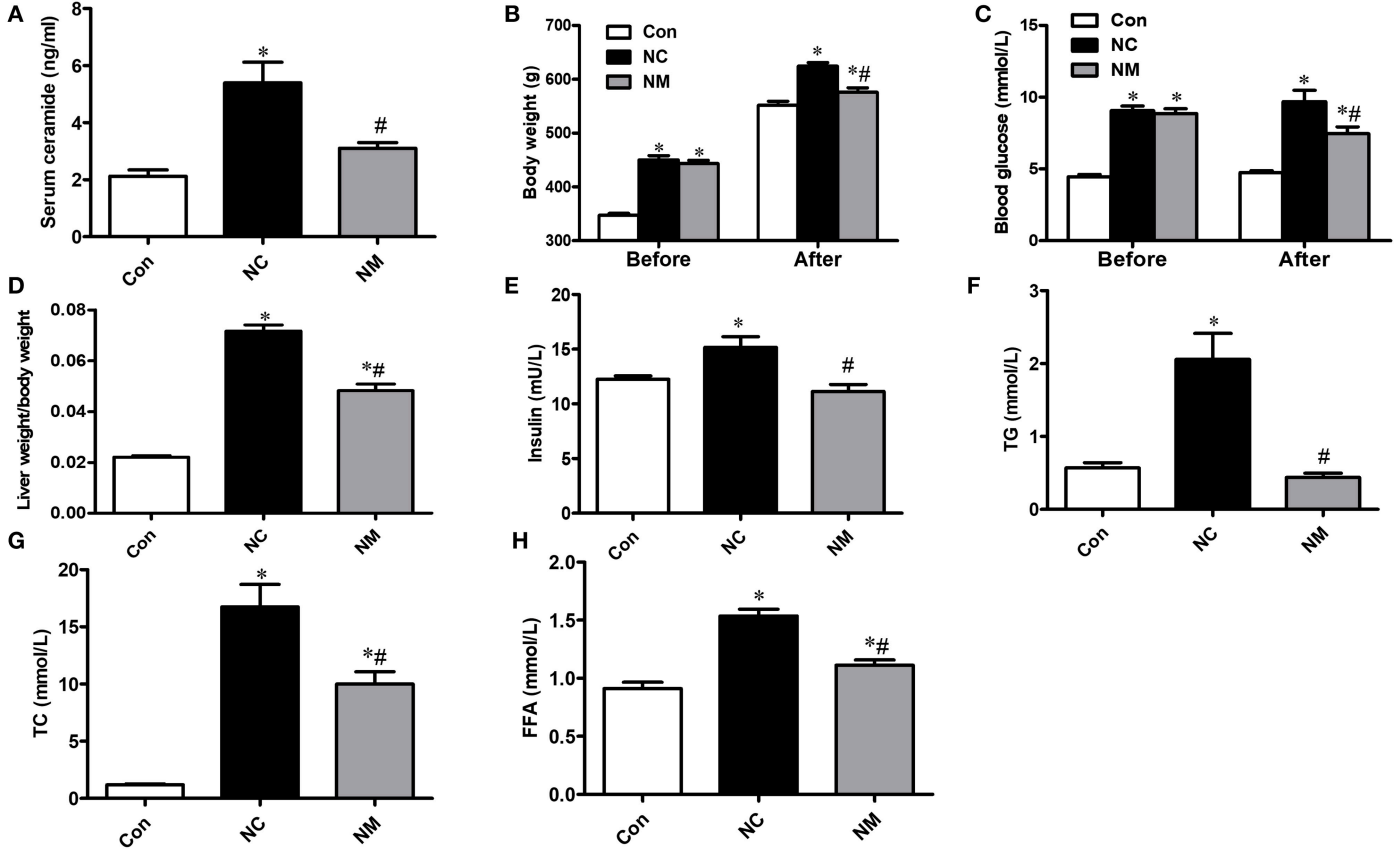
Chronic treatment with myriocin improves metabolic indies and liver damage markers. **(A)** Measurements of serum ceramide (*n* = 6). **(B)** Body weight (before and after intervention) in each group (*n* = 11). **(C)** Liver index (liver weight/body weight) in each group (*n* = 11). Determination of blood glucose (before and after intervention) **(D)**, insulin **(E)**, TG **(F)**, TC **(G)**, FFA **(H)** (*n* = 11). All values are means ± SEM. *P < 0.05, vs. the control group. ^#^*P* < 0.05, vs. the NC group.

As shown in Figure 1A, in the rats fed a high-fat diet, the serum ceramide content was significantly increased, which could be reduced by myriocin treatment. From Figures 1B,C, there was no difference in body weight and blood glucose level between NC and NM group before myriocin intervention. In addition, we observed that body weight (Figure 1B), blood glucose levels (Figure 1C), liver index (Figure 1D), insulin levels (Figure 1E), blood lipid profile (TG, TC, and FFA content, Figures 1F–H), were markedly increased, which could be improved by myriocin treatment.

### Effect of Myriocin on Hepatic Morphology and Inflammation

**Figure 2:**
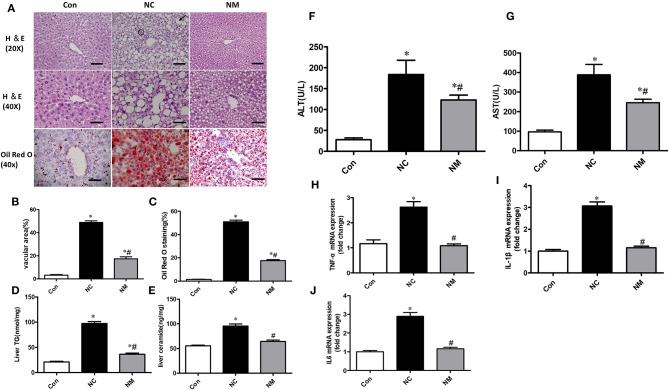
Chronic treatment with myriocin decreases lipid accumulation and inflammation in liver with hepatic steatosis. **(A)** Top row: H&E staining (magnification, ×20), steatosis (bold arrow): lipid droplets are present in hepatocytes. Clusters (aggregates) of inflammatory cells (within circles). Scale bars represent 100 μm. Middle row: H&E staining (magnification, ×40), scale bars represent 50 μm. Bottom row: Oil Red O staining (magnification, ×40), scale bars represent 50 μm. **(B)** Fractional area of the livers containing vacuoles was determined by morphometric analysis (*n* = 5). **(C)** Oil Red O staining area was determined by morphometric analysis (*n* = 5). Evaluation of TG **(D)** and ceramide **(E)** in liver tissue samples (*n* = 11). Measurement of serum ALT **(F)** and AST **(G)** (*n* = 6). RT-qPCR analysis of TNF-α **(H)**, IL-1β **(I)**, and IL-6 **(J)** (*n* = 6). All values are means ± SEM. *P < 0.05, vs. the control group. ^#^*P* < 0.05, vs. the NC group.

From the H&E staining (Figure 2A), we observed that chronic treatment with myriocin decreased the number and size of hepatic lipid droplets that were upregulated by HFD (Figure 2B). Consistently, Oil Red O staining suggested that myriocin treatment led to a lower lipid content in the liver of HFD-fed rats (Figures 2A,C). Consistent with these results, we also observed that myriocin could reduce liver triglyceride (Figure 2D) and ceramide (Figure 2E) content in the HFD-fed animals. In addition, elevated serum ALT (Figure 2F) and AST (Figure 2G) levels induced by HFD were decreased by myriocin treatment.

From the H&E staining (Figure 2A), we also observed that the increased inflammation (the amount of inflammatory cell aggregates) induced by HFD was improved by myriocin treatment. Consistently, RT-qPCR analysis indicated that HFD led to a significant increase in the expression of TNF-α (Figure 2H), IL1-β (Figure 2I), and IL6 (Figure 2J), which was improved by myriocin treatment.

Collectively, myriocin treatment attenuated ceramide accumulation, hepatic steatosis, and hepatic inflammation induced by HFD in rats.

### Effect of Myriocin on Hepatic Morphology and Fibrosis

**Figure 3:**
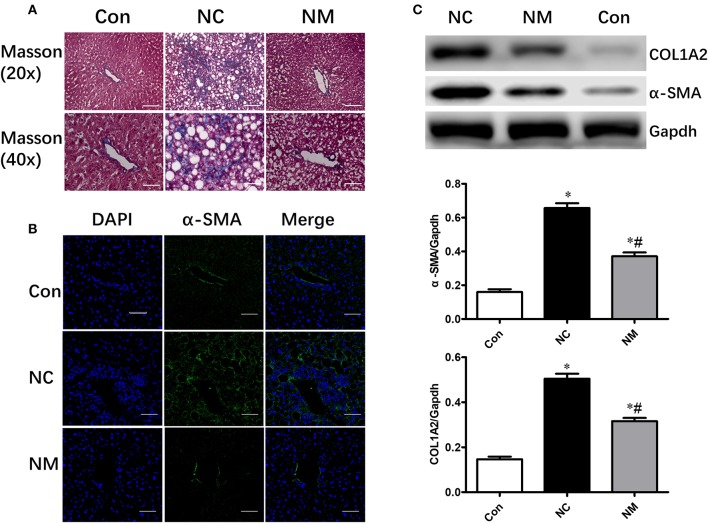
Chronic treatment with myriocin improves hepatic fibrosis. **(A)** Masson staining of liver tissue sections. Top row: Masson staining (magnification, ×20), scale bars represent 100 μm. Bottom row: Masson staining (magnification, ×40), scale bars represent 50 μm. **(B)** IF staining of α-SMA in liver sections. Scale bars represent 50 μm. **(C)** Western blot analysis of α-SMA and COLIA2 in liver samples. All values are means ± SEM, *n* = 5/group. *P < 0.05, vs. the control group. ^#^*P* < 0.05, vs. the NC group.

As shown by Masson's staining, HFD-fed animals exhibited hepatic fibrosis (Figure 3A). However, myriocin treatment significantly attenuated hepatic fibrosis (Figure 3A). Expression of α-SMA (smooth muscle actin), which was examined via an immunofluorescence assay (Figure 3B) and Western blot analysis (Figure 3C), is a reliable marker of the hepatic stellate cell activation that precedes fibrous tissue deposition. The protein expression of α-SMA and COL1A2 was markedly increased in HFD-fed rats. however, only a slight increase was observed in the rats myriocin-treated rats (Figures 3B,C).

### Effect of Myriocin on Hepatocyte Apoptosis in Rats With NAFLD

**Figure 4:**
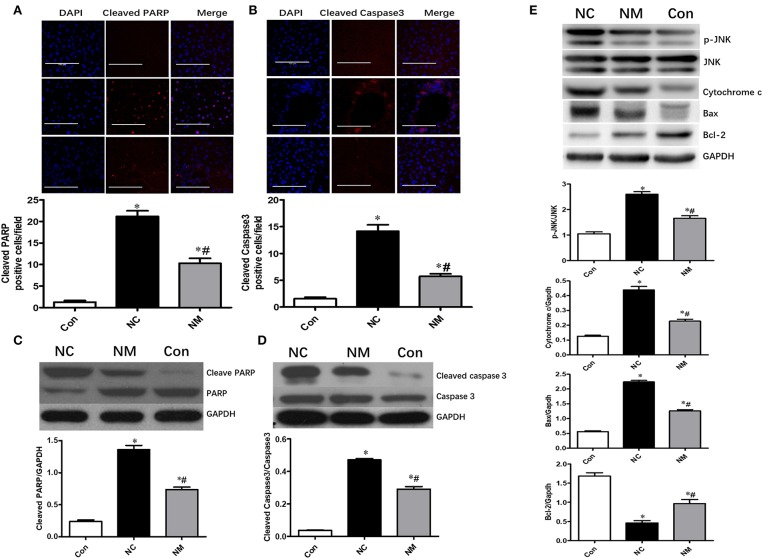
Chronic treatment with myriocin improves hepatic apoptosis by regulating JNK signaling pathway. Cleaved caspase3 and cleaved PARP are valuable markers of apoptosis. Therefore, we assessed hepatic apoptosis by detecting expression of Cleaved caspase3 and cleaved PARP in this study. **(A)** IF staining of Cleaved PARP in liver sections, scale bars represent 100 μm. **(B)** IF staining of Cleaved caspase3 in liver sections, scale bars represent 100 μm. **(C)** Western blot analysis of Cleaved PARP in liver samples. **(D)** Western blot analysis of Cleaved caspase3 in liver samples. **(E)** Western blot analysis of p-JNK/JNK, Cytochrome c, Bax, and Bcl-2 in liver samples. All values are means ± SEM, *n* = 5/group. *P < 0.05, vs. the control group. ^#^*P* < 0.05, vs. the NC group.

Chronic treatment with myriocin resulted in a significant decrease in the levels of cleaved PARP and cleaved caspase 3 in the liver tissue of HFD-fed rats, which became comparable to levels in the control group (Figures 4A,B). Similar results were observed in the change of cleaved PARP and cleaved caspase 3 levels in liver tissue analyzed by Western blot (Figures 4C,D). To further investigate the effects of ceramide on hepatic apoptosis, we detected p-JNK/JNK, cytochrome c, Bcl-2, and Bax expression. As shown in Figure 4E, liver tissue from HFD-fed rats exhibited higher levels of p-JNK/JNK, cytochrome c, and Bax expression than liver samples from normal rats. Additionally, in HFD-fed rats, the expression of p-JNK/JNK, cytochrome c, and Bax was decreased by myriocin treatment (Figure 4E). Myriocin treatment also restored the reduced Bcl-2 expression induced by HFD (Figure 4E). The findings above indicate that ceramide played an essential role in modulating hepatic apoptosis in HFD-fed animals.

## Discussion

The aim of the present study was to investigate the role of ceramide in the development and progression of NAFLD. We observed that HFD induced ceramide accumulation in the livers of rats and resulted in a fatty liver phenotype. Myriocin, a ceramide synthesis inhibitor, markedly attenuated liver lipid accumulation. In addition, hepatic inflammation and fibrosis were markedly ameliorated in myriocin-treated rats. These findings provide clear evidence that ceramide plays an important role in liver lipid homeostasis regulation and in the pathogenesis of NASH. Thus, ceramide synthesis could potentially be a target for the treatment of NAFLD.

Prior studies of obese rodents revealed that ceramide inhibition could improve obesity-related metabolic abnormalities such as insulin resistance, hyperglycemia, and hyperlipidemia (9, 19, 21–23). Prior studies also revealed that blunting *de novo* ceramide synthesis could ameliorate hepatic steatosis in obese animals (6, 11). Consistent with prior results, we observed the same results in the present study. Previous study reported that inhibition of ceramide accumulation can enhance metabolism and energy expenditure (21). This may be an important reason why myriocin can improve weight of obese animals. Inhibition of ceramide synthesis was able to improve insulin resistance from previous studies (9, 18, 21, 22). This may explain why myriocin can improve insulin and blood glucose levels.

Here, we mainly focused on detecting the effects of inhibiting ceramide accumulation in the development of NAFLD. We use a rat high-fat diet fed model to incite NAFLD and hepatic inflammation and use a pharmacologic inhibitor of ceramide synthesis (myriocin) to reduce ceramides systemically and in the liver. The findings of this paper are not novel, while we provide important corroborating evidence that ceramides are important in NAFLD pathogenesis.

However, myriocin treatment markedly attenuated not only lipid accumulation but also hepatic inflammation and fibrosis. In our study, we found that chronic treatment with myriocin resulted in a significant reduction in mRNA levels of inflammatory genes, including IL-1β, IL-6, and TNFα. Meanwhile, the expression of α-SMA and collagen was also markedly attenuated by myriocin treatment in HFD-fed rats. However, the mechanisms by which ceramide promotes the development of NASH remain unclear.

Hepatocyte apoptosis has been considered a key factor of NASH pathogenesis and progression (24). Previous studies showing that ceramide may represent a pro-death molecule suggested that apoptosis may be involved in this process (2, 17). In addition, mice with steatosis that were fed an HFD displayed an increase in hepatic ceramide, which was associated with hepatocyte apoptosis (25). In the present study, we found that hepatic cleaved caspase 3 and cleaved HARP levels were significantly increased in HFD-fed rats but were markedly attenuated by myriocin treatment. Therefore, suppressed hepatic apoptosis may be responsible for improved liver function, inflammation, and fibrosis in myriocin-treated animals.

JNK is one of the most investigated signal transducers in obesity-related conditions (26–28). JNK also plays an important role in the cell stress response including cell proliferation, survival, and death (29). Ceramide can induce cell apoptosis by activation of the JNK signaling pathway (30, 31). Here, we found that p-JNK expression was significantly increased in the liver samples from HFD-fed rats and that the level of expression of these liver samples could be restored by myriocin treatment. The JNK signaling pathway was able to affect Bcl-2 family members. Specifically, JNK regulates the activity of proapoptotic BAX and anti-apoptotic Bcl-2 proteins (32, 33) and facilitates the release of mitochondrial cytochrome c to induce apoptosis (34–37). In addition, ceramide has been reported to activate Bax and inactivate Bcl-2 in the apoptotic pathway (38–40). Our study suggests that myriocin treatment decreased Bax and cytochrome c expression and increased Bcl-2 expression in HFD-fed animals. Briefly, the anti-apoptosis effect in the liver of rats with NAFLD was mediated via the JNK signaling pathway and treated with a ceramide synthesis inhibitor.

In conclusion, myriocin, an inhibitor of *de novo* ceramide synthesis, is considered to have anti-steatosis and anti-fibrosis effects, by activating anti-apoptosis and anti-inflammation mechanisms in HFD-fed NAFLD pathological animals. Therefore, ceramide appears to be a potential target for developing treatment therapies for NAFLD and NASH, which are induced by hepatic apoptosis and inflammation.

## Data Availability Statement

The datasets generated for this study are available on request to the corresponding author.

## Ethics Statement

The animal study was reviewed and approved by the ethical committee of Central South University.

## Author Contributions

MJ carried out the experiment and wrote the manuscript with support from CL. QL contributed to the animal experiment. AW performed the metabolic Index. ML supervised the project. All authors helped shape the analysis, research, and manuscript.

### Conflict of Interest

The authors declare that the research was conducted in the absence of any commercial or financial relationships that could be construed as a potential conflict of interest.
